# The Influence of Civilization upon the Development of Insanity

**Published:** 1853-04-01

**Authors:** A. Brierre de Boismont


### THE INFLUENCE OF CIVILIZATION UPON THE DEVELOPMENT OF INSANITY.

A FRAGMENT. BY DR. A. BRIERRE DE BOISMONT.

(*Translated from the Author's MS.*)

Some fifteen years ago I read a paper before the Academy of Sciences, upon the "Influence of Civilization in the production of Insanity." It consisted of a review of the predominating moral causes of historical researches and statistical documents; but it was deficient in practical experience, which I had not at that period had an opportunity of acquiring.

The question was recently brought before the Medico-Psychological Society, and there caused a discussion which has induced me to express my more matured opinions on the important subject.

The interpretation of the word *civilization* has given rise to countless controversies, and must of necessity be complex, if by it is meant that supremacy in war, in commerce, in industry, in science, or in the arts, by which various nations have in different ages been distinguished. But such is not my idea of the meaning of the word. I take it to denote the totality of immutable principles, (the groundwork of all society,) of the discoveries, and of the knowledge proper to each succeeding age, and transmitted from generation to generation. I consider that civilization being by its nature essentially progressive, is never entirely arrested, although it has the weakness, the infirmity, and the uncertainty inseparable from its human origin; such is the view which will guide me in the following discussion, and I feel that no one can justly accuse me of being opposed to rational progress.

The preceding definition comprises the elements of the only real civilization, that which has for its base the recognition of the unity of God, and for its practice the love of the entire human race, comprising the abolition of all species of slavery, and the elevation of the woman and child from the subjugation under which they laboured in antiquity; in one word, the civilization of Christianity. And is it not evident, even to the most unobservant, that this civilization is now marching with rapid strides to the conquest of the entire world? The genius of man, executing the benevolent intentions of the Deity, has overthrown the barriers of nations, and brought the ends of the earth into such close connexion, that there no longer exists any impediment to arrest it.

Thus having indicated the point from which I start, I will at once proceed to the subject-matter of this article.

There are some ideas which present themselves with such an aspect of truth that we are disposed to receive them at once without any inquiry; they are of the number of those admitted by the common sense of all, which is, as an illustrious writer has said, superior to individual reason. The subject now under consideration was formerly placed by me in the above category, for it seemed to come under the physiological law, which declares that the abuse of the function of a vital organ leads to its fatigue, exhaustion, and impairment; but I have since recognised that intuitive deductions are misplaced in science, and that the circumstantial judgment of physicians requires some definite kind of proof. I have sought for these proofs in the *analysis of the moral nature of man, in statistical documents, and in historical researches*. I shall confine myself for the present to the analysis of man's moral nature, reserving my inquiry into the other two sections for some future occasion.

It is impossible for any person to reflect upon the human mind without being immediately struck with the difference of the two elements of which it consists. On the one part are found ideas which have no immediate derivation from outward things, which seemingly belong to some invisible world, have no actual limit, but, pure children of thought, appear as emanations from, and aspirations towards, that infinite intelligence to which they will some day return. On the other part are seen the sensible images of the world around us, ideas wholly compounded of sensations derived from material objects. These two orders of phenomena comprise all psychological and physical facts. It is easy to understand why a vast majority of mankind gives pre eminence to the first of these two classes, elevating spirit above matter, and putting the moral before the physical. For it is from the moral side only that we can obtain any insight into the mysteries of the invisible world, and can approach the obscure but attractive problems concerning the origin and purport of existence, the freedom of the will, the consciousness of evil, and the immortality of the soul.

Yet it is chiefly by the study of the passions, those mainsprings of human actions, that we can rightly estimate the importance of moral philosophy. Hence the reason why the works of the great moralists have such an enduring interest.

Read once more the letters of Seneca to Serenus, those of St. Chrysostom to Stagirius, the Essays of Montaigne, the Reflections of Pascal, the Characteristics of Theophrastus and La Bruyère, the Maxims of La Rochefoucault, and you will the more readily embrace the immense horizon of moral science. The works that have been written by man upon man preserve their freshness throughout all time, because they treat of the sentiments, the passions, the virtues, and the vices, which are common to humanity in all ages; whilst writings relating to ephemeral matters, the transactions, the events, and the fortunes of the day, can only excite a fugitive interest, and are speedily forgotten.*

* It were easy to multiply examples. M. Aran, in his analysis of a "Treatise on Anatomical and Physiological Chemistry, Normal and Morbid," by MM. Robin and Verdeil, shows that the theory of chemical combustion as a source of animal heat is much shaken by the discovery of an acid in the lungs, that of protein, the base of nitrogenized tissues. The source of fatty matters, which were believed to be drawn from the food; the theory of the formation of urea by the oxidization of the constituent

But I hasten to quit the interesting province of psychological speculation to enter into the more practical one of medicine.

At the commencement of my professional career, chance, as some would say, or, as I say, providence, placed me as domestic physician in great families, and after an apprenticeship of some years, I learnt to form my own opinions upon the neuroses, the gastralgic disorders, the organic diseases of the stomach or lungs, the cerebral affections, &c., &c., attributed to irritation, to inflammation, to asthenia, and other causes equally profound. The secrets of these people so envied by others were laid bare to me, and I was ready to repeat with a celebrated author, "No! happiness has no outward sign." The experience I then acquired has not since been lost upon me.

It was by the observation of two equally instructive cases that I commenced the clinical study of mental maladies. I was called to attend a lady of rank, remarkable for her intelligence, who was suffering from extreme despondency, produced, I was told, by disease of the uterus; in a short time I ascertained that there was really no uterine disorder, but that her melancholy was owing to her having been abandoned by the man whom she had loved for 20 years. This case terminated in a tragical manner. Some months later I accompanied a gentleman of large fortune and considerable attainments in his travels abroad. He had engaged in a hazardous speculation, by which he had risked his entire fortune. Believing himself ruined he attempted to destroy himself, and when convalescent spoke quite unconcernedly of his suicide; "for how," said he, "could I return to Paris, or show myself among my financial friends, after such a foolish action?" A fortunate rencontre cured him, and he returned to resume a high social position.

The character of a man's mind, and the circumstances in which he lives, undoubtedly influence him both in his choice of and his method of investigating a subject. So, actuated by a conviction of the predominance of the moral over the physical elements, without denying or excluding organic influences, I have particularly directed my attention to the research of the psychological causes involved in the production of mental maladies; and I have no hesitation in affirming, as the result of my investigations, that moral agencies produce the greater number of cases of disorders of the mind.

The physician to a private asylum is placed in a different position from the physician to a large public establishment. Although the latter, from his greater choice and greater variety of cases, possesses superior opportunities for the study of symptomatology, and morbid anatomy; can arrive at statistical conclusions, and detail with minute exactitude the different aspects of the disease; he is often destitute of a complete or correct history of his cases, has not the leisure to collect the moral elements, and is frequently obliged to abandon his observations, by the sudden removal of the patient. On the other hand, the resident physician of a private asylum, not having to attend to so great a number of cases, is able to devote a larger share of personal attention to the few under his care. Living among his patients and constantly associating with them, he observes all their movements, their acts, and conversation; he often becomes the friend and confidant of the sufferer, and when self-revelation is deficient, can almost always obtain the information he requires from the relatives and friends, and is thus enabled to unfold the psychological history of each individual case.

The knowledge of the etiology of mental disorders which the intelligent physician thus obtains, by constant personal intercourse with his patients, is much more exact and circumstantial than that which the medical officers of a large public asylum can acquire.

elements of animals, and that of the formation of uric acid by a process of imperfect oxidization, all brilliant theories, and generally accepted, already seem likely to be erased from the catalogue of discoveries.—*Union Médicale*, 25th December, 1852.

It appears to me that the medical officer of an extensive general establishment, when he undertakes to describe the various causes of mental maladies, resembles an historian gravely discussing the causes of wars, of political changes, and of revolutions, which can be accurately known only to the statesmen and persons engaged in them. It is in vain to point to the large residue of unknown causes which the best analysis will leave. I still persist in asserting, that the physician who has more than 200 patients at one time under his care cannot, from mathematical necessity, fully investigate the obscure and hidden causes of insanity.

It is said, that it is often difficult to separate the moral from the physical causes, that the two are so complicated and so closely connected as to render their separation difficult and almost impossible. This is true up to a certain point; but I nevertheless maintain, that in the majority of cases of well-marked insanity which have come under my care, I have been able to trace the origin of the disorder to some passion, sentiment, instinct, or idea,—in other words, to a moral cause.

I could now refer to my own statistical tables, for more than 1200 patients have been treated by me, but I prefer citing the conclusive evidence which men of acknowledged authority have published on the predominance of the moral causes. And first I will quote from M. Parchappe's "Statistical Researches on the Causes of Mental Alienation" (1839), in which he arranges the two orders of causes in the following manner:—

*Comparison of the Moral Causes with the so-called Physical Causes.*

| | Moral Causes. | | Other Causes. |
|---|---|---|---|
| Mania . . . . | 64 in 100 | .... | 36 in 100 |
| Melancholia . . | 77 „ 100 | .... | 23 „ 100 |
| Paralysis . . . | 50 „ 100 | .... | 50 „ 100 |

When M. Moreau de Jonnès brought forward his statistical tables on this subject at the Institute (July, 1843), M. Parchappe addressed a letter* to that learned body, in which he strongly maintained his former opinion, and declared the predominance of the moral causes of insanity over the physical, to be a positive truth added to science.†

In this new inquiry, which comprised the results of his observations on 1476 cases, treated by him in the space of eight years, he stated the relative frequency of the recognised causes to be as follows:—

| | Moral Causes. | | | | | Other Causes. | | | |
|---|---|---|---|---|---|---|---|---|---|
| Men . . . | 222 in 393 | or | 565 | in 1000 | .... | 171 in 393 | or | 435 | in 1000 |
| Women . . | 311 „ 409 | „ | 762 | „ 1000 | .... | 98 „ 409 | „ | 238 | „ 1000 |
| Total . . | 533 „ 802 | „ | 664·6 | „ 1000 | .... | 269 „ 802 | „ | 335·4 | „ 1000 |

The statistical report of the Public Lunatic Asylum for the Department of the Lower Seine, drawn up by Drs. Parchappe and de Boutteville (Rouen, 1845), presents analogous results. In short, M. Parchappe states in all his works, that moral causes taken altogether are by far the most frequently concerned in the production of insanity.

The predominating influence of moral causes is greater upon women than upon men. It is exhibited in the highest degree in connexion with melan-

* "On the Predominance of the Moral Causes in the Production of Insanity."—*Annal. Med. Psych.*, t. ii. p. 365.

† M. Parchappe makes four classes of the determinating causes:—1. Intellectual, affective, and moral causes. 2. Causes which consist in the abuse of these faculties, by the pursuit of intellectual or sensual gratifications. 3. Causes connected with a morbid condition of the organs. 4. External causes; physical, chemical, or physiological.

cholia, is somewhat less marked in mania, and almost disappears in insanity accompanied by paralysis.

The most active causes concerned in the production of mental disorders, generally speaking, are these three: sensual excesses, pecuniary anxieties, and domestic cares. These three causes have a different relative influence on the two sexes, and follows the ensuing order. In man, 1, sensual excesses; 2, pecuniary anxieties; 3, domestic cares. In woman, 1, domestic matters; 2, money matters; 3, love.

That among the exciting causes of insanity the moral should predominate is what we might anticipate theoretically, upon consideration of the mode of life amongst civilized people, in whom the activity of the intellectual faculties is so largely developed.

Before proceeding with the examination of the moral causes, I may be permitted to refer to another argument, which is rightly esteemed of considerable weight; it is the well-known fact that large and populous cities furnish a much higher per centage of insane persons than rural districts.

Among the observations establishing this fact I will take three, which proceed from men who are in the habit of collecting, comparing, and interpreting this kind of statistics, and I select the reports of the Mareville, the Ghent, and the St. Yon Asylums, drawn up by MM. Renaudin, Guislain, Parchappe, and de Boutteville.

According to M. Renaudin, the proportion of insane persons in the department of the Meuse is 1 in every 1468 of the entire population; whilst the proportion of the insane in Nancy, the chief city of that department, is 1 in 500 of the population of that city.

At Ghent, M. Guislain states, there is 1 insane person in every 302 inhabitants, whilst the proportion of the insane in the rural population, amounting to 569,000 inhabitants, does not exceed 1 in 1474.

MM. Parchappe and de Boutteville, who have gone into more exact calculations, make the following statements. During a period of eighteen years (1825 to 1843) the department of the Lower Seine sent to the public asylum 2146 patients, received once or oftener into that establishment. Upon separating and ascertaining the comparative number of lunatics sent to St. Yon from the different districts into which the department is divided, a notable difference is observed.

*Department of the Lower Seine.*

| Districts. | Population, 1841. | No. of Lunatics admitted at St. Yon. | Lunatics in 1000 Inhabitants. |
|---|---|---|---|
| Rouen . . . . . . . . | 248,115 | 1371 | 5·5 |
| Havre . . . . . . . . | 149,427 | 279 | 1·8 |
| Yvetot . . . . . . . . | 142,349 | 201 | 1·4 |
| Dieppe . . . . . . . . | 112,374 | 187 | 1·6 |
| Neuchatel . . . . . . . | 85,246 | 108 | 1·2 |
| Total . . . . . . . | 737,511 | 2146 | 2·9 |

By classifying the admissions according to the population of the districts in which the patients had resided, a still greater disparity presents itself.

| | Population. | | Admissions at St. Yon. | | Proportion in 1000 Inhabitants. | |
|---|---|---|---|---|---|---|
| Town of Rouen . . . . . | 96,002 | | 965 | | 10·05 | |
| „ Havre . . . . . | 27,254 | | 106 | | 3·90 | |
| „ Dieppe . . . . . | 16,443 | | 79 | | 4·80 | |
| „ Elbouf . . . . . | 14,646 | | 53 | | 3·61 | |
| | | 154,345 | | 1203 | | 7·79 |
| Towns and parishes containing from 3000 to 10,000 inhabitants . . . . . . | | 102,375 | | 258 | | 2·52 |
| Parishes containing less than 3000 inhabitants: | | | | | | |
| District of Rouen . . . . . | 107,573 | | 256 | | 2·40 | |
| „ Havre . . . . . | 78,692 | | 85 | | 1·08 | |
| „ Yvetot . . . . . | 124,208 | | 156 | | 1·25 | |
| „ Dieppe . . . . . | 91,954 | | 102 | | 1·10 | |
| „ Neufchatel . . . | 78,454 | | 82 | | 1·04 | |
| | | 480,881 | | 681 | | 1·42 |
| Total of the department . . . . . | | 737,601 | | 2142 | | 2·9* |

It should be stated, that a special asylum for pauper lunatics was established at Havre some years back, since when no patients have been sent from that city to St. Yon. This explains why, in the preceding tables, the number of patients from Havre is so much below what might be expected from the second city of the department. The foregoing calculations plainly prove that the mode of life of the industrious classes in the great centres of population of the department of the Lower Seine, strongly predisposes to insanity; for it is shown that the number of insane persons belonging to the district of Rouen is one-fourth, and even one-third above that of other districts in the same department. And the difference becomes still more striking, upon separating the returns furnished by the principal towns from those of the rural districts surrounding such towns. For example, the entire district of Rouen, which in 1841 contained 248,000 inhabitants, sent, in the space of eighteen years (1825 to 1843), 1371 patients to the St. Yon Asylum; of these 1371 patients, 965 belonged to the city of Rouen, which had 96,000 inhabitants, and 406 to the surrounding district, which comprised 152,000 inhabitants; manifesting a difference in the proportion of 1005 to 267, or nearly 4 to 1. I need hardly remark, that these observations completely contradict the opinion which some writers have advanced, ascribing the relative excess of inhabitants of towns in lunatic asylums to the fact of the large public asylums being usually built in the locality of towns.

But there is also another moral disease which, if not identical with insanity, is commonly so closely allied to it as to afford us a further illustration of the predominance of urban over rural influence in exciting mental disorder: I refer to *suicide*.

Twenty years ago, M. Guerry, in his work on the *Moral Statistics of France*, showed suicide to be more common in large towns and their vicinities, particularly in the departments nearest to Paris. In general, from whatever point on the frontiers of France we start, says this observer, we find the number of suicides increase progressively as we approach nearer to the capital. This progression is particularly noticed in those departments which are traversed by the grand routes leading to Paris from Lyons, Strasburg, Nantes, Bordeaux, &c. If we select Bordeaux, for example, and examine the proportional number of suicides in the successive departments which lie between that city and Paris, and are traversed by the main route, we find, in

* Although this column serves to illustrate the point under consideration, the reader will at once perceive that it has no real value.—Tr.

the department of Charente, 1 suicide for 28,000 inhabitants; of Vienne, 1 in 25,000; of Indre-et-Loire, 1 in 18,000; of Loir-et-Cher, 1 in 14,000; of Loiret, 1 in 10,000; and so on until we come to 1 in 5000 in Seine-et-Oise; and 1 in 3000 for the department of the Seine.

In order to obtain the most precise results, it is advisable to study the proportional number of suicides in the towns and rural districts of the same department. This has been done by MM. Archambault, Etoc Demazy, and Petit. According to the first of these observers, the chief towns of the department of the Meurthe contain only about a fourth of the entire population of the department; and yet in eleven years (1834 to 1845) there were 115 suicides in the towns to 95 in the country, being an approximative ratio of 19 to 5. The results obtained by M. Etoc Demazy are very similar. M. Petit, in his excellent "Statistical Researches on the Etiology of Suicide," has drawn up three tables, with the view of showing the progressive diminution of the suicidal tendency, proportionably to the removal from the influence of towns, which leave no doubt upon the greater prevalence of self-destruction in towns and cities, and entirely refute the opinion of M. Cazauvieilh, who pretended they were equal in the two localities. Here are the mean results taken from M. Petit's three tables:—

| | Mean of Town Population. | Mean of Country Population. | 1 Suicide |
|---|---|---|---|
| 1st series, 29 departments . . | 22 per cent. | 78 per cent. | in 9,918 |
| 2nd series, 29 departments . . | 19 „ | 81 „ | „ 18,984 |
| 3rd series, 28 departments . . | 14 „ | 86 „ | „ 36,721 |

The conclusion to be derived from these facts is evident; for they show that the rate of suicide increases progressively with the increase of the town population, and decreases progressively with the increase of the country population, and that the elevation or depression of the rate of suicide in any particular department above or below the mean standard of suicide in France* is proportionate to the relative amount of town and country inhabitants. When insanity has been investigated in the manner in which M. Petit has investigated suicide, or, more generally, after the mode in which MM. Parchappe and de Boutteville have treated the facts derived from the asylum of St. Yon, we shall have statistical proof of an opinion founded inductively on a philosophic observation of facts, one of the three great sources of human knowledge.

A conscientious observer, who, during thirty years, has laboured in advancing our knowledge of mental alienation, M. Guislain, has in a recent work, which embodies the result of his long and varied experience, added new documents on the predominance of the moral causes of insanity to those which he published in 1826 concerning the same subject. As the conclusion of his researches he declares, that the source of morbid conceptions must be sought for in the internal nature of ideas. In order to arrive at a precise knowledge of the causes so considered, he recommends that we should exclude idiots, imbecile persons, cretins, cases of temporary delirium, hysterical persons, and epileptical, from the summary of moral causes. To these exceptions we would also add delirium tremens, although often tracing its remote cause to some depressing emotion.

These distinctions established, M. Guislain clearly propounds the problem of the cause of insanity, so debated in these times: he says, that in 66 per cent. of the cases admitted into the establishment he directs, he has discovered mental, psychical, or moral influences; and in 100 cases attributable to moral causes exclusively, he found 65 dated from some reverse, especially reverse of fortune, and 35 to domestic troubles. M. Delasiauve fixes the prevalence of

* In 1846 the mean rate of suicide in France was 1 in 11,420 inhabitants.—Tr.

moral causes at a still higher rate, for he considers that 80 in 100 cases of insanity are determined by some depressing and exhausting passion or emotion.

Moral causes are, then, according to Pinel, Esquirol, MM. Guislain, Parchappe, Delasiauve, ourselves, and many others, the chief agents in the generation of insanity, which opinion, however, by no means excludes the share which may be justly allotted to physical causes, as is seen by the care which all the preceding investigators have taken to enumerate and analyze them. That there should be great disparity of opinion about the relative predominance of moral causes may be attributed, as M. Guislain observes, to the circumstances which so often hinder the discovery of such causes, the insufficiency and inexactitude of investigation, the want of close personal intimacy with patients, and to errors derived from the ignorant or interested misrepresentations of the patient's friends.

"If you desire examples," says this competent physician, "let me relate to you the following cases, which have come under my own observation. A lady consulted me about her husband, who was labouring under an attack of insanity, assuring me that she could in no way explain it, and knew of nothing that could have induced it. When restored to reason, the husband informed me that his wife's misconduct had been the sole cause of his illness. A young man, of timid, reserved, and pious disposition, was admitted into my establishment in a state of insanity, produced, it was told me, by excessive study. I suspected a certain injurious habit, and remorse of conscience at not being able to overcome it, and I subsequently received a confession from my patient which entirely confirmed my suppositions. An old gentleman was brought to me in a state of imbecility, which his nieces, who accompanied him, ascribed solely to his advanced age. Further information, obtained from disinterested persons, revealed one of those domestic dramas which are, it is to be feared, too common; the nieces had endeavoured to force the old gentleman into making certain testamentary bequests, and on his refusal had employed so much violence and ill-treatment towards him as to reduce him to the condition above described."

In these ideas I entirely concur, and some years ago I expressed myself to the same effect in the "Medico-Psychological Annals" as follows:—

"If you ask why the real, the moral cause of insanity so often escapes our inquiry, I answer that it is because it is so often intentionally concealed. How can you expect the relatives or friends of your patient to be candid? Shall a parent say, 'Behold my son, whose misconduct is my despair; whose ill behaviour is the misery of my life?' or, 'Here is my daughter, whose irregularities and indiscretions I am unable to control?' or, 'This is my son-in-law, who commits excesses which will, I fear, soon lead to some terrible catastrophe;' or shall a child say, 'I bring you my father; he is squandering our substance in riot and debauchery?' or shall a husband tell you, 'My wife is guilty of the grossest outrages towards me, but I am reluctant to expose her on account of our children and my own reputation?' and a thousand similar complaints!"

Like M. Guislain, I am embarrassed in my choice of appropriate illustrations of my subject; but I select the following instance as an example in support of the foregoing general conclusions, which are themselves but the simple expressions deduced from the analysis of facts. A foreign lady recently called on me, accompanied by her mother, to consult me about her mental disorder, which was characterized by periodical attacks of maniacal excitement coming on suddenly, without any premonitory symptoms, and passing off entirely at the end of one or two days. After informing me of the above particulars, she proceeded to give me an explanation of the circumstances to which she attributed her state of mind. "I have no taste," said she, "for the pleasures of society, so I live almost constantly alone, employing myself with different kinds of work, and indulging in no amusements. This sort of life, combined with certain vexations, no doubt exerts considerable influence on my mental condition." After she had been some time under my care, I received a confidential communication from a distinguished professional brother, which completely rectified the circumstantial statements of the mother and daughter. My informant disclosed to me that this lady had been a chief actor in a domestic drama of unusual depravity. Whilst still innocent, she had become the victim of the extraordinary villany of a near relation, who had artfully seduced her, not from any passion or attachment for her, but actuated by a belief still too commonly entertained, that he could thus get rid of a shameful disease with which he was infected. The lady's health suffered; she was made aware of the terrible injury she had sustained; all her affections were embittered; she became melancholy and morose, and retired as much as possible from society, which had become hateful to her. Whilst in this condition of mind, her family, with the view of saving appearances, and stopping the spread of certain rumours detrimental to its honour, persuaded or compelled her to consent to be united to a gentleman, unacquainted with her history. A marriage contracted under such circumstances could have but one result; the injured and distracted husband revealed his misfortune, and sought reparation for his wrongs; but so artfully was the lady's cause conducted by herself and family, that he soon found himself enveloped in a web of contradiction and recriminatory accusation, which he had neither the patience nor the address to unravel, so that in the end he was regarded as the offending party; the lady obtained an act of separation, and the husband entered the army. After these circumstances had been revealed to me, I could not wonder why my patient had sought to conceal from me the real history of her case.

It would but weary the attention of my readers if I were to insist further on the predominance of the moral causes of insanity. The examples I have quoted show how great an effect injured honour, reverse of fortune, disappointed affection, and domestic trouble have in deranging the balance of the human mind. And surely an inquiry into the motives, the speech, and the actions of mankind, although undertaken mainly with the view of ascertaining the causes of their aberrations, is a legitimate portion of moral science, and a fit subject for moral analysis.

I come next to treat of another influence which governs all moral causes; from which they arise and flow in a thousand different ways; this influence, but partly understood, and but half appreciated, is moral suffering. Twelve years ago, in reviewing a work by M Leuret, I took the influence of moral suffering in the production of insanity as the theme of my argument. (" Gazette des Médécins Praticiens," 1840.) In that paper I cited several remarkable instances, some of which I think I may venture to repeat.

" What a catalogue insanity presents! Kings, legislators, sages, philosophers, all are found upon its lists. And poets too, what a space they occupy in the roll! I one day visited a town in Italy; I passed rapidly before cathedrals, churches, palaces, public monuments, fountains, statues, all interesting, perhaps, but I did not stay to inspect them, for my entire attention was directed to the spot towards which I was hastening. At length I stopped before a low arched door-way inside an iron railing. Nothing could be more sad and sombre than the aspect of this retreat, yet the names of Byron, Chateaubriand, Lamartine, Delphine Gay, and many others, showed that some mighty interest was connected with this spot. It was, in fact, the cell in which Tasso had been confined for seven years, and his hard couch and iron ring still remained in their places. On seeing these things I fancied I could hear the illustrious poet ringing the death-knell of Chatterton, of Collins, of Gilbert, and a host of brothers, lost and abandoned like himself.

" Political life also affords its contingent. Lord C. rules the most powerful nation in the world; at his command the armies of Europe take the field, and irresistible fleets plough the seas in every direction: but all his powers cannot procure him internal tranquillity, or peace of mind; consumed by chagrin, or rather by remorse, he seeks in vain for happiness; his sad condition is irreparable, until at length reason gives way, and a tragical death terminates his sufferings.* Lord C., instigated by an insatiable cupidity, commits the most revolting crimes; at length the outcries of the thousands he has plundered arrive to the ears of parliament; an inquiry is instituted, but remorse has already seized him, he becomes a prey to the direst despair, and terminates a glorious career of success by his own hand.† P.‡ assumes the reins of government at a moment when the throes of popular convulsion yet shake the state, and by his wisdom and energy succeeds in restoring public tranquillity; but the indifference of his contemporaries, and a foresight of the ingratitude of posterity towards him, irritate and offend him; his intellect becomes confused, insanity manifests itself, and he dies, struck down by the terrible

* Dr. Brierre de Boismont has not been rightly informed of the circumstances which produced Lord Castlereagh's temporary insanity. That illustrious statesman served his country with a pure and elevated patriotism, which left him no cause for remorse; but his mind gave way under the immense labour imposed upon it, and the constant anxiety inseparable from his high and responsible office. An incident of domestic life *is said* to have accelerated the progress of his mental disorder; but there is no reasonable doubt of his case being one of a class unfortunately too frequent among us—derangement produced by the over-work and excessive exercise of the mind. The late Sir Robert Peel, writing of Lord Castlereagh in 1839, observes, "He overcame practical difficulties which would have appalled and overwhelmed almost every other contemporary statesman." In these few words we see a confirmation of the opinion we have above expressed.—Tr.

† Dr. Brierre de Boismont is also in error concerning Lord Clive. There is not the slightest ground for believing that he suffered from an evil conscience; on the contrary, he is known to have looked back upon his Indian career with a just and excusable pride, and he strenuously defended every act of his Indian administration, both before Parliament and elsewhere. The actual circumstances of his suicide are so well told in Macaulay's admirable essay, that we trust our readers will pardon us for quoting the passage: "Clive was now secure in the enjoyment of his fortune and his honours. He was surrounded by attached friends and relations, and he had not yet passed the season of vigorous bodily and mental exertion. But clouds had long been gathering over his mind, and now settled on it in thick darkness. From early youth he had been subject to fits of that strange melancholy 'which rejoiceth exceedingly and is glad when it can find the grave.' Whilst still a writer at Madras, he had *twice attempted to destroy himself*. Business and prosperity had produced a salutary effect on his spirits. In India, while he was occupied by great affairs—in England, while wealth and rank had still the charm of novelty, he had borne up against his *constitutional misery*. But he had now nothing to do, and nothing to wish for. His active spirit in an inactive situation drooped and withered like a plant in an uncongenial air. The malignity with which his enemies had pursued him, the indignity with which he had been treated by the committee, the censure, lenient as it was, which the House of Commons had pronounced, the knowledge that he was regarded by a large portion of his countrymen as a cruel and perfidious tyrant, all concurred to irritate and depress him. In the meantime, his temper was tried by acute physical suffering. During his long residence in tropical climates, he had contracted several painful distempers. In order to obtain ease, he called in the help of opium; and he was gradually enslaved by this treacherous ally. To the last, however, his genius occasionally flashed through the gloom. It was said that he would sometimes, after sitting *silent and torpid* for hours, rouse himself to the discussion of some great question, would display in full vigour all the talents of the soldier and the statesman, and would then sink back into his melancholy repose. On the 22nd of November, 1774, he died by his own hand. He had just completed his forty-ninth year." It is a remarkable fact, that Lord Castlereagh and Lord Clive both committed suicide in the same manner—by a stab in the neck with a common penknife.—Tr.

‡ Casimir Périer became insane whilst exercising the functions of prime minister to Louis-Philippe, and died immediately afterwards, May 16, 1832.

scourge of human reason. B. fulfils with credit and distinction the high diplomatic missions confided to him. He acquires a place in history by his honourable share in a negotiation of the first importance; but mortifications coming from high quarters worry and annoy him; he gets tired of life, and puts an end to a wearisome existence. 'The death of poor B.,' wrote a young prince who has left a cherished memory, 'affected me greatly, and I think must have affected you in a similar manner. I say nothing about the painful impressions produced at N., where the law on suicide is so severe; what touches me most is the inquiry into the causes which led him to commit the fatal act B. was not ill; he executed his design with perfect sang froid and resolution. I have received letters from M. and others which remove all doubts from my mind. He was sorely irritated against the father; and spoke strangely to F. about him. The authority which the father exercises over all is of so inflexible a character, that when a minister compromised by his connexion with us cannot overcome it, he has no other resource but suicide.' " *

M. Guislain, who has completely developed the theory of moral suffering, shows by a great number of well selected and well argued instances, that it is often the initial phenomenon of insanity, and marks the prodromic period of the disorders. This action of moral suffering results from that excessive exaltation of sensibility which M. Cerise has so well termed "*emotivité*." Among civilized people, a great activity of the moral sentiments exists, and great susceptibility of the affections, which induced M. Guislain to say that many cases of insanity have their origin, not in the mind but in the heart. To feel is the ardent desire of many natures, and a female saint strongly expressed this longing when she chose for her device the words—"*Ou souffrir ou mourir*."

The epochs in which moral suffering is most prevalent, when it assumes all forms, and attacks all ranks, but especially the classes whose sensitiveness is continually stimulated, are decidedly those which are distinguished by the greatest perfection in the luxurious arts, in literature, and in those scenic illusions which partly supply the craving for emotions which the universal satiety of real life at such times fails to afford.

Hearts unmanned by luxury and corruption, although eager to indulge in factitious woe, have not sufficient courage or energy to patiently endure the real miseries of life. When the conquerors of the world had reached the climax of their power, repose became so difficult for them, that a folded leaf on their bed of roses was sufficient to banish sleep from their eyelids; ill befel the unlucky slave who then vexed their nerves—the vivarium was his grave. Such were the times most fruitful in moral sufferings; disgust of life and ennui were universal, and very many of these masters of the earth sought a refuge from satiety in suicide, or tamely submitted their necks to the imperial executioner. Compare their condition with that of their ancestors during the first centuries of the Roman empire; consider the position of the early settlers in New England, their courage and resignation, their simple manners embellished by sincerity and religious faith, and ennobled by the sense of duty; recal to memory the savage virtues of the native Indians, their patient endurance of privations, and their stoical contempt of death inflicted by the most horrible tortures; and then say if sensibility, and the consequent capacity for suffering, is not in direct proportion to the perfection of luxury, and the exercise of the intellect, in other words, to the progress of civilization.

* The diplomatist in question was M. Bresson, French ambassador at Brussels in the eventful year 1831, negotiator of the matrimonial alliance between the Duke of Orleans and the Grand Duchess Helen of Mecklenbourg-Schwerin (1837), and of the famous Spanish marriages (1846). A few months after the last event he committed suicide at Naples, in consequence, it was suspected, of his having incurred the displeasure of King Louis-Philippe, "the father" alluded to in the letter above quoted.—Tr.

Ultimate analysis brings us therefore to moral suffering as the starting point of insanity in the majority of instances. In the battle of life all must suffer, but especially those naturally endowed with a nervous, passionate, and susceptible organization. When suffering has arrived at its extreme intensity, when it admits neither of suspension nor alleviation, and the faculty of resistance is extinct, human consolation is a vain pretence, for the mind cannot receive it, and despair offers but two issues—suicide or madness.

The sufferings of the heart, so well known to the psychological physician, that he could become their best interpreter if his labours and habits of life allowed him to undertake the task, have been eloquently treated of by the great moralists of preceding ages. But it is notably by modern writers that this subject has been systematically discussed, and its universality and deep importance revealed. "In these days," says M. E. de Montégut, "it matters not in what spot human misery lies hidden, or in what corner injustice is perpetrated; an unseen eye discovers, and an unknown voice proclaims the wrong inflicted, the suffering endured. Not now, any more than in former days, does good prevail; evil still reigns triumphant, but evil can no longer be stifled or concealed." "We strive in vain," lately wrote M. Paul de Molènes, "to overcome the sadness of these times; our age is still that of Werther, of Manfred, and of René. We can never be made to smile frankly and without reserve. Whoever says the contrary is in error. There has not been one heart among us during the last sixty years but was born a prey to *ennui*, regret, and melancholy."

The present epoch is not only consumed by *ennui*, but is also a prey to a multitude of moral maladies, among which I may name the universal confusion of ideas, the general weariness, and the entire disenchantment concerning all we were proud of and adored. We feel the institutions we were vain of having moulded fall to pieces under our hands. Full of uncertainty and doubt, agitated by sinister presentiments, we seek a refuge in what I fear I must call domestic selfishness, and are willing to make the greatest sacrifices to procure a short period of repose. Our literature—and the literature of a people is the image of its morality—tells but one tale; the double wrong inflicted upon humanity, in the body and in the soul; on our moral and our physical nature. A few days since I read a book which has been compared to a shrill and piercing note, vibrating through space, and jarring all the tenderly sensitive strings of the human heart; a book written in tears and blood, the subject of which is not drawn from the barbarous ages, nor from the dark annals of antiquity, but describes scenes of horror constantly recurring in our own times, and the sufferings of millions of victims day by day renewed in the land which styles itself the Land of Liberty, and which prides itself on its Christian faith, and its strict observance of the Sabbath. Everywhere is heard the cry of pain, and a clever woman has well said: "The human soul is an instrument which vibrates in unison with all the emotions; joy produces a short and rapid sound, which is soon extinguished; but the tone of grief is loud, deep, and prolonged." This fragment has already become too long, or I could extract from the literature, or rather, from the history of philosophy, arguments sufficient to reply to the objection often raised, that we have not sufficient statistical information to enable us to come to any precise conclusions concerning the moral condition of epochs much anterior to our own, or to warrant a satisfactory comparison. Moreover, I have already stated, that I have reserved the historical department of my subject for separate treatment, so I will confine myself at present to a few short remarks.

History would be an arid waste, destitute of interest, if we were compelled to measure the errors, the aberrations, or the progress of the human mind by the aid of statistics only; for, notwithstanding the recent improvements of that science, it does not always furnish correct information, even in inquiries which seem best suited to its capabilities, and is certainly of little value in the appreciation of moral facts. Then, again, it is so extensible as to yield in all directions, and accommodate itself to the most opposite views; so that we often see the same data employed in the deduction of diametrically opposed results. Nevertheless, I am far from rejecting the aid of statistical inquiry, but I regard it merely as an useful auxiliary, and I attach much more importance to the literary records of different nations. You remark that each succeeding age has left some sign of its intellectual and social progress, so that history enables us to trace the march of the human mind across the expanse of ages. In the youth of Greece and Rome, we see it full of life and vigour, asserting its freedom, the base of all philosophy, and resisting the encroachments of tyrannical authority; then, involved in the universal darkness which attended the invasion of the barbarians and the fall of the Roman empire, it remains immovable during the slow destruction and renovation of society under the Merovingians; presenting few signs of vitality during the first 500 years of the monarchy, except in the steady progress of ecclesiastical denomination, it at last bursts forth into new life and growth at the commencement of the sixteenth century.

Two quotations will suffice to present us with the means of estimating the state of the human mind at two distinct epochs, and of deciding by the light of reason, and without the assistance of figures, the question now before us, of the different degrees of mental excitability, or rather of moral sensibility, in different centuries. In the time of Charlemagne, says M. Guizot ("De la Civilisation en Europe," t. ii.), men had but few ideas, and those ideas extremely restricted. Social intercourse was rare and limited. The horizon of the mind, like that of life, had narrow confines; under such condition a great society is impossible.

This rarity of ideas is also noticed in M. A. de Broglie's work, "Sur le Moyen Age et l'Eglise Catholique." A small number of simple ideas, expressed in poor, rude, unfinished language, sufficed to kindle the zeal of ardent hearts, and enlighten the dawning imagination. Compare the state of the public mind in its infancy with that of our own times, when countless periodicals daily give us news from the remotest corners of the earth, when railroads enable us to overrun Europe in a few days, and the shortest life may suffice to witness three or four revolutions, each accomplished in a different view.

Some questions have been raised about the caution exercised by the writers we have quoted in the choice of their materials; their political principles are open to discussion, but no one has a right to deny their scientific talent or literary probity.

Nor would the proofs be less decisive, if for each of these periods we should relate the history of the celebrated person who has amassed them.

If I had needed any further confirmation of my views on this matter, I should have found them in M. St. Marc Girardin's remarks on some of the moral disorders of modern times.

After having sketched in a few concise and vigorous strokes the character of the French nation, showing that it has not changed since the time of Cæsar, he paints in strong and lively colours the disorders which revolutions let loose in the brains of sober citizens, which agitate the ambitious, and influence even persons most desirous of repose. When the reader has completed his perusal of this moral clinic on these three sections of society, he will understand the optimist and pessimist character of the first, the credulous and speculative disposition of the second, the cosmopolitan indifference of the third, one hundred times better than if he resorted to statistics to supply him with an approximative valuation of their comparative numbers.

To these considerations I venture to add another, which is also of some importance. Society, in its ascertained movement, does not always proceed in the same manner. Either it rests upon faith, or it operates by doubt. In the first condition, authority governs; in the second, individual reason. These conditions obtained in the Roman Empire as well as in the Christian world. Now, of these two states, the one most likely to develope insanity is that which admits of individual opinion, and not that of faith and authority. Hence the necessity of specifying the precise character of the civilization of different epochs; for the term civilization has a vague signification, unless the attendant circumstances are explained. There lies the gist of the question, or rather the light best adapted to discover it.

I have yet one observation to make before I conclude these reflections. Moral causes are to be usefully studied, not merely in relation to the etiology of mental disorders, but also with reference to their particular treatment. At the end of an article printed in the "Annales d'Hygiène," 1839, I appended this note: "In a future number I shall speak of the employment of moral means in the treatment of insanity, and perhaps I may be able to add something to the labours of others in this department."

Up to the present time I have not been able to fulfil my intention, but I have put the views which I intended to publish into practical execution. Whilst observing the miserable victims of melancholia annihilated, as it were, by mental suffering, I saw that there was something to be done beyond the prescribing of medicines, and I was naturally led to employ the resources and consolations of domestic life. I will not boast of having effected a greater number of cures by these means than may be obtained by others, but I can say that by these means I have consoled and restored many unfortunate persons.

The advantages of the domestic treatment of the melancholic form of insanity are now too well known to require any exposition here, so I will make but one remark upon it, which refers to the comparative facility of its application. The moral treatment does not demand any extraordinary qualities or superior powers on the part of those engaged in it; discrimination, judgment, and, above all, kindness, are what it requires for its success. The man of genius may sometimes obtain a striking recovery by exceptional methods; but it is the kind-hearted physician, who treats his patients as if they were his children, who most frequently succeeds in effecting a permanent cure.

In a psychological point of view the question of the influence of civilization in the development of insanity appears to me to be conclusively settled; for since it has been shown that the moral causes of mental disorders exceed all others in frequency, and that moral suffering is their most common origin, it follows that these two causes may be expected to arrive at their greatest intensity in those epochs when moral sensitiveness is most actively developed. Of course I speak only of the present time, and do not presume to decide for the future.

*Summary.*—The analysis of man's moral nature places the predominance and pre-eminence of the psychological or moral element over the physical beyond all doubt.

The dominion exercised by the moral over the physical, observable in many diseases, is particularly obvious in insanity; and moral causes are certainly the causes before all others which have the most marked influence on the production of mental disorders.

Statistics prove, incontestably, that moral causes altogether are the most frequent determinating causes of mental derangement. The same conclusion is derived from the analysis of the moral life of civilized nations.

The conflict of opinions about the relative predominance of moral causes is to be attributed to imperfect information, the impossibility of making a careful psychological examination of the numerous patients admitted into the large public asylums, the limited amount of personal intercourse with such patients, and the uncertain period of their stay.

All moral causes converge towards some primitive source of moral suffering, which exerts an universal and almost permanent influence.

The effect produced by moral suffering is in direct proportion to the sensitiveness of the sufferer.

The epochs which are marked by the greatest development of sensitiveness are those in which the civilizing force is impaired by luxury, by scenic illusions, and by an imaginative literature; and such epochs are, consequently, most fertile in moral disorders.

The literary history of a people, by which we are enabled to trace the social and intellectual movement of each succeeding age, seems preferable to mere statistical deductions, and is more to be relied on in investigating the state of the human mind at different epochs.

As moral suffering is, in the majority of instances, the remote or proximate cause of insanity, so the moral treatment is naturally the best adapted to the cure of the disorder, and is, what may be correctly styled, the remedy *par excellence.*

From the preceding considerations we may come to the conclusion, that a practical analysis of man's moral nature, as we see it in operation, is the only method of satisfactorily deciding the question of the effect of civilization in the development of insanity.

---

